# Pediatric Age Palm Oil Consumption

**DOI:** 10.3390/ijerph15040651

**Published:** 2018-04-01

**Authors:** Lorenza Di Genova, Laura Cerquiglini, Laura Penta, Anna Biscarini, Susanna Esposito

**Affiliations:** Pediatric Clinic, Department of Surgical and Biomedical Sciences, Università degli Studi di Perugia, 06132 Perugia, Italy; lory.digenova@gmail.com (L.D.G.); laura.cerquiglini@ospedale.perugia.it (L.C.); laura.penta@ospedale.perugia.it (L.P.); anna.biscarini@ospedale.perugia.it (A.B.)

**Keywords:** palmitic acid, palm oil, saturated fatty acid, breast milk, cardiovascular disease, obesity, overweight

## Abstract

Palm oil is widely used in the food industry for its chemical/physical properties, low cost and wide availability. Its widespread use has provoked an intense debate about whether it is a potential danger to human health. In a careful review of the scientific literature, we focused on nutritional characteristics and health effects of the use of palm oil with regards to children, seeking to determine whether there is evidence that justifies fears about the health effects of palm oil. Our review showed that palm oil represents a significant source of saturated fatty acids, to which scientific evidence attributes negative health effects when used in excess, especially with regards to cardiovascular diseases. However, to date, there is no evidence about the harmful effects of palm oil on the health of children. Nevertheless, palm oil has possible ill health effects linked to its composition of fatty acids: its consumption is not correlated to risk factors for cardiovascular diseases in young people with a normal weight and cholesterol level; the elderly and patients with dyslipidaemia or previous cardiovascular events or hypertension are at a greater risk. Therefore, the matter is not palm oil itself but the fatty-acid-rich food group to which it belongs. The most important thing is to consume no more than 10% of saturated fatty acids, regardless of their origin and regardless of one’s age. Correct information based on a careful analysis of the scientific evidence, rather than a focus on a singular presumed culprit substance, should encourage better lifestyles.

## 1. Introduction

Palm oil is characterized by chemical/physical properties, low cost and wide availability and for these reasons is largely used in the food industry r [1,2,3,4,5,6]. However, there has been an intense debate about whether it is a potential danger to human health [1,7,8,9,10,11,12,13,14,15,16]. In 2016, the European Food Safety Authority (EFSA) produced a document on the risks related to the consumption of refined vegetable oils. In particular, the Panel on Contaminants in the Food Chain (CONTAM) warned of the presence of substances formed during the production process, particularly when vegetable oils are refined at high temperatures (approximately 200 °C); the oils undergo partial hydrolysis of triglycerides with glycerol oxidation, leading to the formation of 3-monochloropropanediol (3-MCPD) and 2-mono-chloropropanediol (2-MCPD) [1]. The highest levels of these compounds were observed in palm oil, but the majority of refined vegetable oils contain considerably large quantities. Interestingly, while refined palm oil might contain high amounts of MCPD, raw palm oil might be considered healthy.

In 2012, the Codex Alimentarius recommended the use of technological adjustments to reduce the levels of 3-MCPD in the finished product. In 2013, the International Agency for Research on Cancer (IARC) stated that there was no evidence to suggest that 3-MCPD is not genotoxic, and the same agency had already classified glycidol in the 2a group (i.e., probably carcinogenic to humans) in 2000 [2]. In a careful review of the scientific literature, we focused on nutritional characteristics and health effects of the use of palm oil with regards to children, seeking to determine whether there is evidence that justifies fears about the health effects of palm oil.

## 2. Materials and Methods

This article provides an overview of papers regarding palm oil and child health in the past 20 years. A PubMed search indexed for MEDLINE and EMBASE was undertaken to identify studies in children and adults using the terms “palm oil”, “palmitic acid”, “saturated fatty acid”, “health”, “children”, “cardiovascular diseases, “cholesterol”, “postprandial lipemia”, “diabetes”, “inflammation”, “cancer”, “overweight” and “obesity” as key words. Only English-language articles were reviewed. The references of the selected articles were consulted for additional contributions that might be relevant. The date of our last search was August 2017, and the period covered was approximately 20 years. 

## 3. Palm Oil

### 3.1. Historical Notes, Biochemistry and the Role of Palm Oil in the Food Industry

The first historical records related to human use of palm oil date back to five thousand years ago, when it played an essential role in the mummification process in Egypt [3]. Its use in manufacturing, including artisanal and industrial production, subsequently spread to Western Africa, Asia and, later, Europe. Its use for food purposes became considerable in the nineteenth century, when it became one of the most widely used vegetable oils in the food industry [3].

Palm oil is produced in tropical areas (Indonesia and Malaysia) and is derived from palm nuts. Its fat has a solid consistency at room temperature because of its elevated percentage of saturated fatty acids. The crude form is known as red palm oil for its coloration, which is derived from elevated concentrations of α- and β-carotenoids. In addition, the native form is rich in other antioxidants, including phytosterol and vitamin E (in the form of α-tocopherol and tocotrienol), which protect the cellular membrane and influence platelet aggregation, modulating the synthesis of thromboxane. Palm oil was also shown to be an excellent oil source for delivering vitamin A in countries with high prevalence of vitamin A deficiency and malnutrition [15,16]. It could be demonstrated that the stability of vitamin A is much higher in palm oil than in soybean oil, which makes palm oil a superior vehicle in fortification programs. In Europe, palm oil is used after refinement, which strips it of carotenoids and similar antioxidant compounds. 

Palm oil should not be confused with palm kernel oil, derived from palm seeds, which has a significantly higher concentration of saturated fatty acids [3]. Globally, palm oil is used as follows [4]: (1) 80% in the food industry (i.e., oil for frying, margarine, baking products, many processed foods); (2) 19% in cosmetics, soaps, lubricants, pharmaceuticals, lacquers and similar products; (3) 1% for the production of biodiesel. Crude palm oil is made up of almost 100% lipids, nearly all in the form of triglycerides. Saturated fatty acids (predominantly palmitic acid) constitute approximately 45% of its total fatty acids. The balance between saturated and monounsaturated plus polyunsaturated fatty acids (approximately 1:1) is nutritionally favourable. 

The metabolic effects of palm oil are influenced by the stereospecific distribution of the different fatty acids: palmitic acid is esterified in the sn-1 and sn-3 positions of the glycerol in 87% the cases and in the sn-3 position approximately 12% of the time, while the unsaturated fatty acids (oleic and linoleic) are predominantly esterified in the sn-2 position. This particular distribution affects their intestinal absorption: fatty acids that are esterified in the sn-1 and sn-3 positions are substrates of pancreatic lipase and are released in the intestinal tract. Fatty acids that are esterified in the sn-2 position are instead absorbed efficiently, similar to monoglycerides, after hydrolysis of the fatty acids at sn-1 and sn-3 [5].

The more efficient absorption of fatty acids esterified in the sn-2 position also explains the different absorption of the various fatty acids in breast milk versus bovine milk. Palmitic acid is the predominant saturated fatty acid in the human diet and is the most common saturated fat in breast milk. In contrast to bovine milk, breast milk contains a large quantity of palmitic acid esterified in the sn-2 position. This makes the absorption of palmitic acid easier for the nursling, which favours rapid growth in the first months of life [6,7].

Palm oil undergoing interesterification procedures has a nonselective distribution of saturated fatty acids in the sn-3 binding position of glycerol. After interesterification, palmitic acid is no longer selectively excreted in the faeces and is absorbed more efficiently owing to its presence in the sn-2 position. In the literature, palmitic acid esterified in the sn-2 position seems not to have direct effects on postprandial lipemia, glucose metabolism or insulin compared with palmitic acid esterified in sn-1 or sn-3 [8,9].

Potential critical objections to palm oil are derived mainly from the use of oil exposed to many heating and cooking cycles in industry and catering, since those conditions are conducive to the formation of oxidation products, which are unfavorable for the cardiovascular system [7,10].

Currently, the food industry pays special attention to industrial transformation processes such as temperature, sudden changes in heat and pressure, and critical contamination levels of glucose fatty acids (GE), 3-MCPD, 2-MCPD and their esters to guarantee the quality of their products [1]. The same production companies are required to respect the environment by adhering to projects for the sustainability of palm oil, working together in national palm oil alliances. 

### 3.2. Possible Alternatives: Advantages and Disadvantages

Food lipids are made up of glycerides, principally triglycerides. Saturated fatty acids, especially myristic, palmitic and stearic acids, are the main compounds of mammal fats, while polyunsaturated fats are prevalent in fish oils [12,17,18]. Cooking oils derived from vegetables contain lesser quantities of saturated fats. Oleic acid prevails in olive oil and linoleic acid in corn, sunflower, grape seed, and safflower oil. Coconut and palm kernel oil have notably higher quantities of saturated fats than other oils. Linoleic and linolenic acids are found in abundance in soybean oil [18]. Some vegetable oils, such as margarine, undergo many processes of hydrogenation that allow the polyunsaturated fatty acids to assume trans structures, losing their profile of metabolic safety (Table 1) [4,12]. 

The possible alternatives to palm oil use in the food industry are the following [19]: (1) saturated animal fats, solid or semi-solid at room temperature, such as butter, lard or tallow: they have been gradually eliminated or substituted for their unfavourable health consequences; (2) saturated vegetable fats, solid at room temperature, such as cocoa butter (the most expensive fat), coconut and palm kernel: their use is limited owing to their correlation with increased plasmatic low-density lipoprotein (LDL) cholesterol levels; (3) other vegetable oils, rich in mono/polyunsaturated fats and fluid at room temperature, from fruits (olives), seeds (sunflowers), legumes (peanuts, soy and especially rapeseed) and grains (corn, rice), despite their nutritional qualities, they are disadvantageous in industrial use, owing to both their costs and their limited shelf life. 

In conclusion, the substitution of saturated fats with unsaturated ones could be favourable from a nutritional viewpoint, but it involves decreases in crunchiness, palatability and some preservation properties, causing a series of disadvantages, especially in the preparation of bakery products. It is extremely important to recommend that the consumer pay attention to what is reported on the product’s label: the use of palm oil in processed products is often linked to other ingredients, such as animal and vegetable fats. The standards for food labelling require information about the ingredients but not their precise quantities. Generally, however, nutritional labels report information on the content of saturated fats. 

## 4. Dietary Fats: Nutritional Recommendations

Currently, nutritional recommendations suggest that fat intake should not exceed 30–35% of total calories, with a maximum of 10% from saturated fats. US guidelines for 2015 to 2020 define the target range of fat intake as between 30 and 40% of calories in early childhood and between 25 and 35% starting at the age of 4 years, keeping the consumption of saturated fats under 10% of total calories [20]. 

In 2011, the French Agency for Food, Environmental and Occupational Health & Safety (ANSES) stated that there is no evidence of benefits associated with fat consumption of less than 35% of total calories; furthermore, the risk associated with a fat consumption of up to 40% of calories has not been clearly documented [3].

In 2012, the Nordic (Scandinavian) Nutrition Recommendation raised the maximum value of acceptable lipids intake to 40% and stated that a level of fat consumption below 20% of total energy would increase the risk of low absorption of other macro- and micronutrients. The limit for saturated fats remains unchanged at 10% [21].

In 2014, the Italian Society of Human Nutrition (SINU) compiled the fourth revision of the Dietary Reference Values (Livelli di Assunzione di Riferimento di Nutrienti ed energia per la popolazione italiana (LARN)) for the Italian population, defining an interval of 20–35% of calories from fat as a benchmark for maintaining one’s health without compromising the adequate absorption of essential fatty acids and fat-soluble vitamins. In particular, fat intake should not exceed 35–40% of total calories in children up to 3 years old, with a maximum 10% of calories from saturated fat for all age ranges [17]. The INRAN-SCAI 2005–2006 survey, the most recent investigative nutritional sample of the Italian population, shows that both total and saturated fat intake are slightly above the recommended levels (36% for total fats, 11% for saturated fats) in children between 3 and 18 years old [22]. The intake of saturated fats from foods containing palm oil in Italy was recently better defined by the Italian Institute of Health, reworking data supplied by INRAN-SCAI. According to this document, the absorption of saturated fatty acids from palm oil in foods is higher during breastfeeding and weaning [4]. Moreover, bakery products represent significant sources of saturated fats from palm oil, especially in younger children. This document also shows that the primary nutritional source of saturated fats in Italy is dairy, followed by oils (mainly olive oil, whose consumption should not, however, be reduced), fresh and processed meats and, lastly, baked goods [23]. 

When recommendations are compared with consumption, the actual Italian diet does not seem well balanced: smoked meats are eaten four times a week instead of twice a week; the amount of milk and yogurt is less than the recommended amount, while cheese consumption is higher than the desirable level. Moreover, legumes are almost absent. Therefore, it would be advisable to raise the intake of some products such as legumes, milk and yogurt in place of red meat, processed meat and cheeses. These changes could reduce the proportions of both total fat and saturated fat in the diet, decreasing them from 36 to 34% and from 11 to 10% of total calories, respectively, and would also diminish the level of salt consumption [3].

## 5. Palm Oil and Saturated Fatty Acids: Effects on Children

### 5.1. The First Six Months of Life

Dietary fats represent the main energy source during the first years of life, and they perform a crucial role in nurslings’ growth and development, especially for premature neonates, because they are among the constituents of the nervous and retinal tissue.

The newborn must immediately switch the main source of calories form glucose (placenta) to fats (breast milk) [24]. The composition of human milk is adapted to the needs of newborns and thus represents the gold standard on which to base infant formulas. In the absence of breast milk, formulas play a crucial role in ensuring the correct intake of nutrients.

In human milk, unsaturated fatty acids, including optimal percentages of essential fatty acids (α-linolenic and linoleic), are more prevalent than saturated fatty acids. Saturated fatty acids, 50% of which consist of palmitic acid, account for 40% of total fat. Moreover, the particular stereospecific distribution of the different fatty acid triglycerides guarantees advantageous absorption. The differences between breast milk versus bovine milk are essentially qualitative; this is the reason why bovine milk is enriched with oleic acid and saturated lauric, myristic and palmitic acids [24]. In breast milk, palmitic acid is esterified at the sn-2 position, while in formulas it is often found in the sn-1 and sn-3 positions, leading to less absorption and more solid faeces, which are typical in artificial breastfeeding.

Vegetal oils that are rich in saturated fatty acids, including some tropical oils (such as palm and coconut oils), are used for formulas in order to increase their energetic value [25]. In addition, the inter-esterification process induces favourable effects in infant formula, as the absorption of palmitic acid increases [25]. Recently, Sanders has demonstrated that palmitic acid in sn-2 can reduce postprandial lipemia, confirming the joint FAO/WHO study of 2008 [8,26]. A valid nutritional alternative for premature newborns, in the absence of breast milk, is the human milk bank, which has been shown to be a better alternative than formula milk. 

### 5.2. Six Months–Two Years 

Weaning is an important dietary change: the caloric contribution of lipids decreases from 50 to 35–40% at 24 months owing to a progressive increase in the amount of carbohydrates, which will continue into adulthood [27]. To date, there is no evidence of any link between dietary fat consumption before 2 years of age and later health conditions, particularly for non-transmissible chronic pathologies such as diabetes, metabolic syndrome or cardiovascular disease [28]. It is difficult, however, to determine the specific metabolic effects of a particular fatty acid and how much it influences the lipid profile in subsequent life phases. It is still difficult to understand the roles of various factors (i.e., gender, degree of excessive weight, distribution of body fat, level of motor activity, composition of macronutrients in the diet) on the optimal dietary lipid profile [29]. 

### 5.3. Three Years–Eighteen Years 

From 3 years old onwards, the nutritional recommendations regarding fats are the same as those for adults. It is necessary to pay greater attention to one’s lifestyle during these years, as the consumption of baked goods, snacks, cookies and chips is especially widespread in this age range [4]. Desserts and other such products contain refined oils, enhanced flours, chemical yeast, synthesized vitamins, sugars, high salt content, toxic products deriving from high-temperature cooking, and palm oil as well. This is the reason why palm oil cannot be considered the only culprit for the ill health effects of this junk food [30]. 

## 6. Palm Oil and Saturated Fatty Acids: Different Metabolic Aspects of Health Risks

### 6.1. Effects on the Lipid Profile

In the 1970s, the Seven Countries Study showed that the dietary consumption of saturated fats correlated to total cholesterolaemia and LDL, known factors in coronary risk [29]. Subsequent studies confirmed the connection between the levels of saturated fats and an increase in LDL cholesterol. The fatty acid composition of palm oil consists of high amounts of palmitic but also oleic acid. Consumption of oleic acid is considered as healthy. On the contrary, due to the fact that palm oil contains palmitic acid, which is a saturated fatty acid, it was extrapolated that palm oil should give rise to elevated total cholesterol and LDL cholesterol levels. However, the effects of palm oil on the lipid profile emerge from a meta-analysis of studies on the replacement of palm oil with mono-unsaturated fatty acids, polyunsaturated fats, myristic and lauric acid or trans-unsaturated fats: such substitutions do not induce variations in the LDL/HDL ratio [31]. The effect of palm oil on LDL cholesterolaemia seems to be considerably less than expected [32]. In conclusion, palm oil does not exert significant effects on the lipid profiles of young people or of people with normal cholesterolaemia. 

### 6.2. Correlations with Cardiovascular Diseases

The guidelines suggest that saturated fat consumption be restricted to 10% of total calories. However, there is no scientific evidence that supports the real benefits of such a limit for the reduction of cardiovascular disease onset; it is still controversial [33]. A meta-analysis that focused on the relationship between saturated fats and cardiovascular risk did not identify any significant link [34]. It must be considered that cardiovascular disease development is multifactorial; therefore, it is difficult to understand the real role played by palm oil alone. Further studies on this potential risk are needed before drawing any conclusion.

### 6.3. Oncological Risk

The implications of palm oil use for oncological risk are not well known. The consumption of palm oil does not have a relevant impact on the risk of cancer [35]. Protective effects in relation with free radicals and anti-inflammatory action are also carried out by micronutrients, such as carotenoids, tocopherol, and tocotrienols, which are abundant in palm oil. Nevertheless, these compounds are absent in the palm oil usually used in the food industries in Europe. In conclusion, there is no evidence that standard consumption of palm oil in packaged foods plays a relevant role in increasing or decreasing the risk of cancer.

### 6.4. Correlation with Obesity 

A recent study (LIPOGAIN) [36,37] conducted on young, healthy, average-weight and slightly overweight subjects (Body mass index (BMI) 18–27 kg/m²) highlights that the consumption of desserts prepared with palm oil causes a more atherogenic lipoprotein profile, a doubling of visceral fat and a significant increase of hepatic steatosis compared with the use of sunflower oil.

Such effects are probably connected with the consumption of fatty acids in general and not palm oil in particular, as similar results were observed with the use of butter in the place of palm oil [38]. However, definitive data in this regard do not exist in the literature [39].

### 6.5. Correlation with Mortality Rate

Recently, the scientific literature has paid attention to the relationship between saturated fat in diets and the mortality rate. According to a meta-analysis published in 2015 [12], the correlation between the consumption of saturated fats and all-cause mortality is zero. This consideration is confirmed by the PREDIMED study [40] conducted in a Mediterranean population at high cardiovascular risk. Unsaturated trans fats are the only fatty acids associated with an increased risk. In literature, different studies have evaluated the effect of various dietary fats on coronary mortality: in particular, in 2016 The NutriCODE group showed that a favourable impact (−11%) on coronary mortality would be obtained by adequately increasing polyunsaturated-fat omega-6 consumption, while the reduction of saturated fats within 10% indicated by the guidelines would only translate to a reduction of 1% in the same coronary mortality. On a global scale, the reduction of saturated fats would produce results of modest importance [41]. The evaluation of the effects of palm oil on cardiovascular health and on all-cause mortality is more complex for the lack of specific data. The only relevant indirect data is the absence of correlation between the levels of palmitic acid absorption and coronary risk in a study of American nurses, at least for consumption up to 10% of total calories [42,43].

## 7. False Myths

After careful evaluation of the literature, we reviewed the portrayal of palm oil role in mass media. Biscuit packaging seems to project the notion of better quality by quite simply adding the label “without palm oil”, suggesting that the absence of palm oil makes any food more desirable and safer for one’s health. This conception a mistake. To date, there is no evidence that palm oil is a threat to be avoided; it is more relevant to understand the amounts written on the label, paying attention to the quantity of saturated fats contained in the food, rather than which fat is used. Eliminating palm oil does not guarantee the greater quality of the product. Therefore, it is necessary to debunk some myths and state some truths.

It is false that palm oil is dangerous for one’s health. The balance between saturated fatty acids and polyunsaturated fats does not make it more damaging than other fats. The warning to not exceed total saturated fat limits remains valid [3].

It is false that palm oil is especially bad for children. Palmitic acid is fundamental in infantile nutrition [44,45,46,47].

Regarding the fact that palm oil contains toxic substances, it is true that according to the EFSA, toxic substances like GE, 2-MCPD and 3-MCPD can be found in palm oil. These contaminants derive from the transformation processes used: they are not part of the oil’s makeup, but rather consequences of production process errors [1].

It is false that substituting palm oil with other oils is an advantage for the consumer. It depends on the fat with which it is substituted and on the total percentage of saturated fats contained in the product [3].

Moreover, it is only partially true that palm oil is bad for the environment. Palm oil is one of the most profitable resources of tropical regions, especially in Malaysia and Indonesia, allowing for economic development. However, environmental damage does exist: from 1990 to 2014, Indonesia lost an extended forestland area with considerable damages inflicted by deforestation. Therefore, in 2013 The Palm Oil Innovations Group set the goal of improve respect for the environment, avoiding deforestation and mistreatment of workers [48]. 

## 8. Conclusions

Palm oil is a widely used ingredient in the food industry. It represents a significant source of saturated fatty acids, to which scientific evidence attributes negative health effects when used in excess, especially with regards to cardiovascular diseases. They are contained in palm oil added to food during industrial transformations, but they are also present in other foods that naturally contain them (milk and its derivatives, eggs, meat). The required intake of fatty acids is greatest during the first few years of life and decreases with age. However, the consumption of saturated fats in the western population is higher than the suggested amount for the prevention of cardiovascular diseases, especially in children from 3 to 10 years of age. 

To date, there is no evidence of harmful effects of palm oil on the health of children; in fact, palmitic acid has been to be necessary in the first years of life, as it is one of the main components of breast milk. Nevertheless, palm oil has possible ill health effects linked to its composition of fatty acids: its consumption is not correlated to risk factors for cardiovascular diseases in young people with a normal weight and cholesterol level; the elderly and patients with dyslipidaemia or previous cardiovascular events or hypertension are at a greater risk. Therefore, in the context of a balanced diet, rich in foods with normal saturated fatty acid content, it is necessary to repeat the importance of limiting the intake of saturated fats foods with a high saturated fat content, in order to respect the quantity recommended in the nutritional guidelines (10% maximum value of total calories). 

Following the request on the eventual toxicity of palm oil as a dietary ingredient coming from the Directorate General for Hygiene in Food and Nutrition of the Ministry of Health, the Italian Ministry of Health has recently drawn up its scientific and technical opinion. The document concludes that no food or ingredient is definable as toxic in and of itself [4]. The eventual negative effects on health are to be measured on the basis of exposure levels.

In conclusion, the matter is not palm oil itself but the group of fatty-acid-rich foods to which it belongs. The most important thing is to consume no more than 10% of saturated fatty acids, regardless of their origin and regardless of one’s age. Correct information based on a careful analysis of the scientific evidence, rather than a focus on a singular presumed culprit substance, should encourage better lifestyles. 

## Figures and Tables

**Table 1:** Different compositions of palm oil and other fats.

	Palm Oil	Soybean Oil	Canola Oil	Sunflower Oil	Olive Oil	Coconut Oil	Butter	Margarine
Saturated fatty acids	45–55	11–21	2–8	10–16	9–26	55–75	49–51	28
Lauric acid C12:0	0–0.5	<0.2	<0.2	<0.2	<0.05	44–51	2–4	<0.2
Myristic acid C14:0	0.5–2	<0.2	<0.2	<0.2	0.05	13–18	8	<0.2
Palmitic acid C16:0	39.5–47.5	8–13	1–5	5–8	7.5–20	8–10	21	20
Stearic acid C18:0	3.5–6	3–6	1–2	4–6	0.5–6	0.5	9	5
Monounsaturated fatty acids	38–45	17–26	56–65	15–26	56–87	7–10	24	30–32
Oleic acid C18:1n-9	36–44	17–26	55–62	15–25	55–83	5.5–7.5	21	16–20
Polyunsaturated fatty acids	9–12	54–72	26–32	62–70	4–22	2–4	2–4	18–20
Linoleic acid C18:2n-6	9–12	50–62	18–22	62–70	3–21	<2.5	1–2	16–18
Alpha-linolenic acid C18:3n-3	<0.5	4–10	8–10	<0.2	<1	<1	1–2	1–2
